# Supplementary figures and images for: Dipeptidyl dipeptidase-4 inhibitor recovered ischemia through an increase in vasculogenic endothelial progenitor cells and regeneration-associated cells in diet-induced obese mice
Source: PLoS One. 2019 Mar 19;14(3):e0205477. doi: 10.1371/journal.pone.0205477 (PMC6424405; doi:10.1371/journal.pone.0205477)

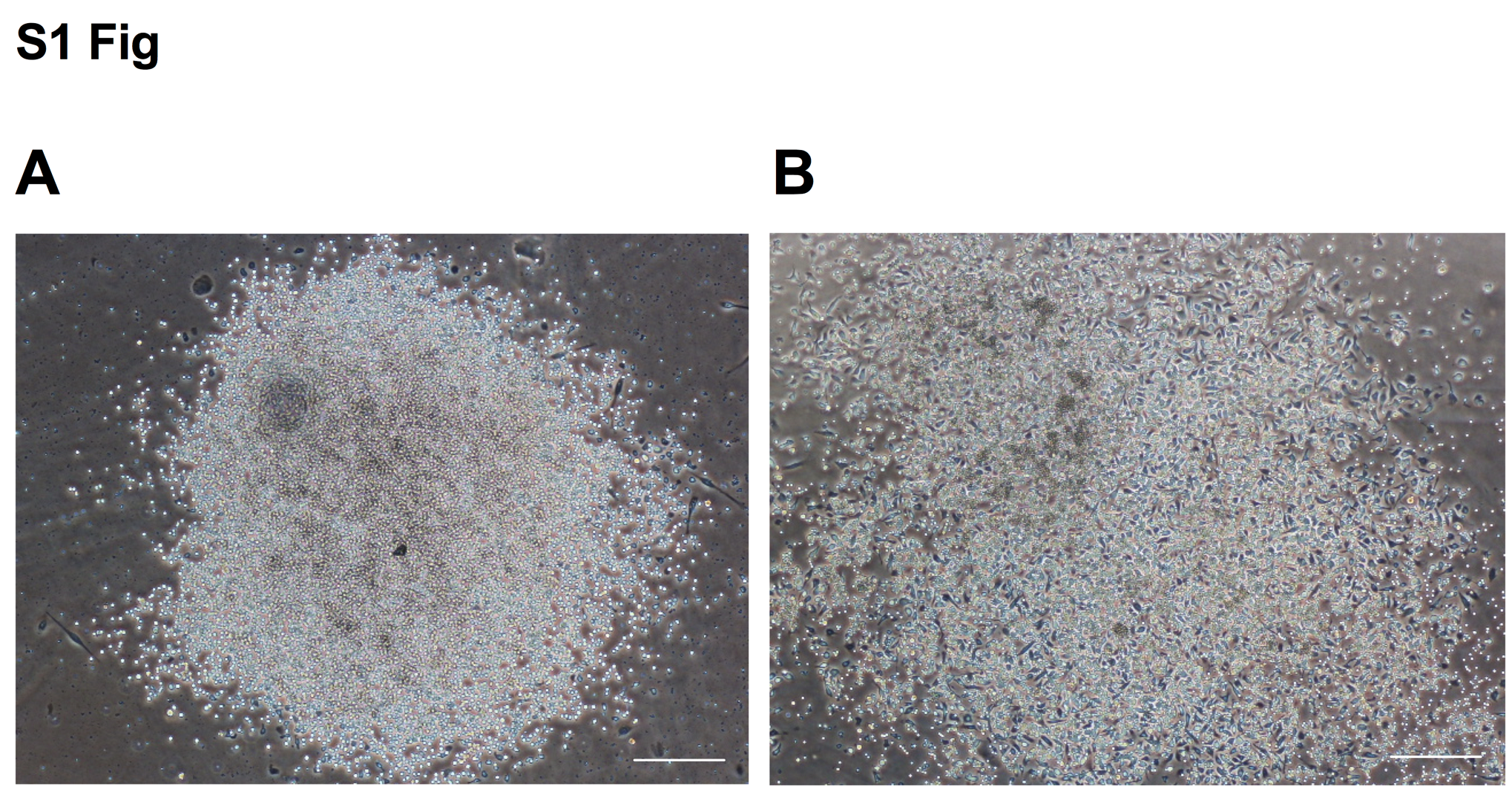

Supplement: S1 Fig — (TIFF) [file pone.0205477.s001.tiff]

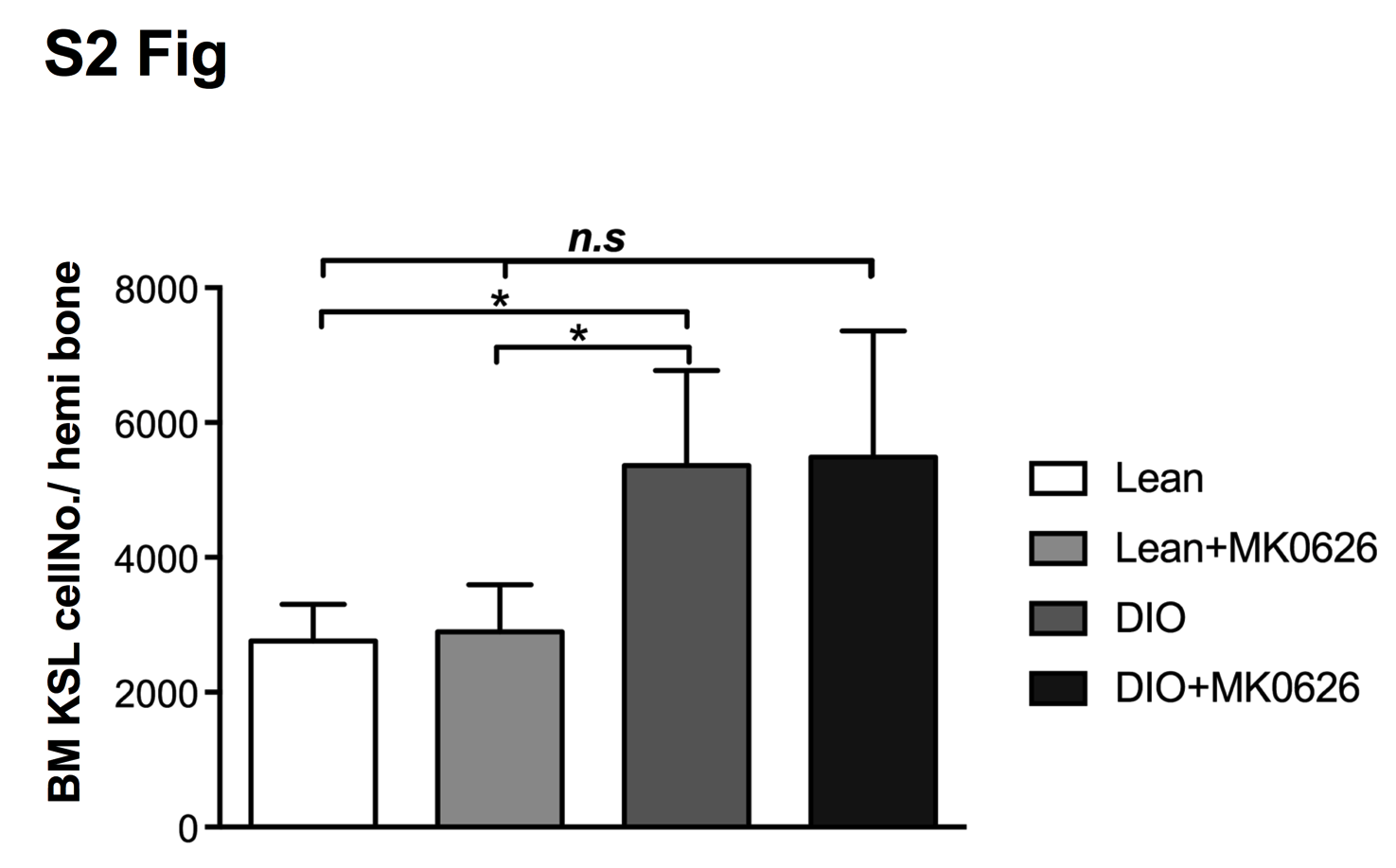

Supplement: S2 Fig — (TIFF) [file pone.0205477.s002.tiff]
